# Statin Therapy and Mortality in HIV-Infected Individuals; A Danish Nationwide Population-Based Cohort Study

**DOI:** 10.1371/journal.pone.0052828

**Published:** 2013-03-04

**Authors:** Line D. Rasmussen, Gitte Kronborg, Carsten S. Larsen, Court Pedersen, Jan Gerstoft, Niels Obel

**Affiliations:** 1 Department of Infectious Diseases, Odense University Hospital, Odense, Denmark; 2 Department of Infectious Diseases, Copenhagen University Hospital, Hvidovre, Denmark; 3 Department of Infectious Diseases, Aarhus University Hospital, Skejby, Denmark; 4 Department of Infectious Diseases, Copenhagen University Hospital, Rigshospitalet, Copenhagen, Denmark; University of Alabama at Birmingham, United States of America

## Abstract

**Background:**

Recent studies have suggested that statins possess diverse immune modulatory and anti-inflammatory properties. As statins might attenuate inflammation, statin therapy has been hypothesized to reduce mortality in HIV-infected individuals. We therefore used a Danish nationwide cohort of HIV-infected individuals to estimate the impact of statin use on mortality before and after a diagnosis of cardiovascular disease, chronic kidney disease or diabetes.

**Methods:**

We identified all Danish HIV-infected individuals (1,738) who initiated HAART after 1 January 1998, and achieved virological suppression within 180 days. Date of first redemption of a prescription of statin was obtained from the Danish National Prescription Registry. We used Poisson regression analysis to assess adjusted mortality rate ratios (aMRR). First, time was censored at date of virological failure (VL >500 copies/ml). Second, time was not censored at virological failure. All analyses were adjusted for potential confounders.

**Results:**

In the analyses confined to observation time without virological failure (+ censoring) statin therapy was associated with a non-statistically significant reduced rate of death (aMRR 0.75; 95% CI: 0.33–1.68). No difference was observed in the analysis with no censoring (aMRR 1.17; 95% CI: 0.66–2.07). Use of statin seemed to reduce mortality in individuals after a diagnosis of comorbidity {(+ censoring: aMRR: 0.34; 95% CI: 0.11–1.04), (−censoring: aMRR: 0.64; 95% CI: 0.32–1.29)}. No difference in rate of death could be detected before first date of diagnosis of comorbidity {(+ censoring: aMRR: 1.12; 95% CI: 0.34–3.62), (−censoring: aMRR: 0.90; 95% CI: 0.28–2.88)}.

**Conclusion:**

Statin therapy might reduce all-cause mortality in HIV-infected individuals, but the impact on individuals with no comorbidity seems small or absent. An unambiguous proof of a causal relation can only be obtained in a randomized controlled trial, but the sample size predicted may be prohibitive for its conduct.

## Introduction

HMG CoA Reductase Inhibitors (statins) are cholesterol-lowering drugs used extensively in the primary and secondary prevention of cardiovascular disease [1]. Recent studies though have suggested that statins possess cholesterol-independent or pleitropic effects including diverse immune modulatory and anti-inflammatory properties [2], [3]. A wide range of beneficial effects have thus been hypothesized.

It is well established by several large clinical trials [4]–[9] that statin therapy can reduce the risk of coronary and cerebrovascular events, and decrease mortality due to coronary artery disease. During the last years a large number of cohort studies have investigated the impact of statin therapy on mortality for a wide range of other medical conditions. As such, studies on chronic obstructive pulmonary disease [10]–[11], sepsis [12]–[14], multiple sclerosis, non-ischemic heart failure and rheumatoid arthritis [15]–[17] have indicated potential protective effects of statin therapy with reduction in both all-cause as well as cause-specific mortality. However, as small negative studies are absent (publication bias), and very few results from randomized controlled trials (RCTs) are present, the evidence in general seems poor.

Recently the potential role of statins in HIV infection has also been subject of a debate. Despite successful suppression of HIV-replication with highly active antiretroviral therapy (HAART), HIV-infected individuals may have persistent inflammation, which can lead to a higher risk of age-associated non-AIDS morbidity [18] and mortality. As statins might attenuate inflammation [2], [19]–[22] statin therapy could potentially have beneficial effects on mortality in HIV-infected individuals beyond the known impact on cardiovascular disease. In a recent study, Moore et al. [23] found that, in HIV-infected individuals on HAART, statin therapy was associated with a lower risk of all-cause mortality (Adjusted Mortality Rate Ratio (aMRR): 0.33; 95%CI: 0.14–0.76). Evaluation of effects of drugs in cohort studies may be substantially hampered by unmeasured confounding and confounding by indication [24]. However, currently there are no data from RCTs to prove this possible effect in HIV-infected individuals. As RCTs are expensive and time consuming, Moore et al. proposed that further observational cohorts studies should investigate the potential protective effect of statin therapy in HIV-infected individuals [23]. We therefore conducted a nationwide cohort study using similar strategies for data analysis to determine the impact of statin therapy on mortality in HIV-infected individuals. We further applied additional strategies of data analysis.

## Methods

### Setting

As of 1 January 2010 Denmark had a population of 5.5 million, with an estimated HIV prevalence of 0.1% among adults [25]–[26]. Treatment of HIV infection is restricted to eight specialized centers, where patients are seen on an outpatient basis at intended intervals of 12 weeks. Antiretroviral treatment is provided free-of-charge. During the follow-up period of the study, national criteria for initiating HAART were HIV-related disease, acute HIV infection, pregnancy, CD4 cell count <300 cells/µl, and, until 2001, plasma HIV-RNA >100,000 copies/ml.

### Data sources

We used the unique 10-digit civil registration number assigned to all individuals in Denmark at birth or upon immigration to link data from the following registers:

#### The Danish HIV Cohort Study (DHCS)

DHCS, which has been described in detail elsewhere [27], is a nationwide, prospective, population-based cohort study of all Danish HIV-infected individuals treated at Danish hospitals since 1 January 1995. DHCS is still ongoing, thus consecutively enrolling new HIV-infected patients and immigrants with HIV infection. Data are updated yearly on demographics, vital status, AIDS defining events, dates of and information on initiation of or changes in antiretroviral treatment. CD4 cell count and viral loads (VL) are extracted electronically from laboratory data files.

#### The Danish Civil Registration System (DCRS)

DCRS, established in 1968, is a national registry which stores information on vital status, residency, and migration for all Danish residents [28].

#### The Danish National Hospital Registry (DNHR)

DNHR, established in 1977, records data on all patients discharged from non-psychiatric hospitals in Denmark. Diagnoses are coded according to the *International Classification of Diseases* [8^th^ revision (ICD-8) until December 31 1993 and 10^th^ revision (ICD-10) thereafter] [29]–[30]. Since 1995 data on outpatients and emergency patients have been included.

#### The Danish National Prescription Registry (DNPR)

DNPR, established in 1994 by the Danish Medicines Agency at the National Board of Health [31], records individual-level data on all redeemed prescriptions dispensed at Danish community pharmacies with complete data since 1 January 1995. The register includes variables related to the drug user, prescriber, pharmacy and the dispensing.

### Study period

The study period was 1 January 1998 through 31 December 2009.

### Study population

**Figure 1:**
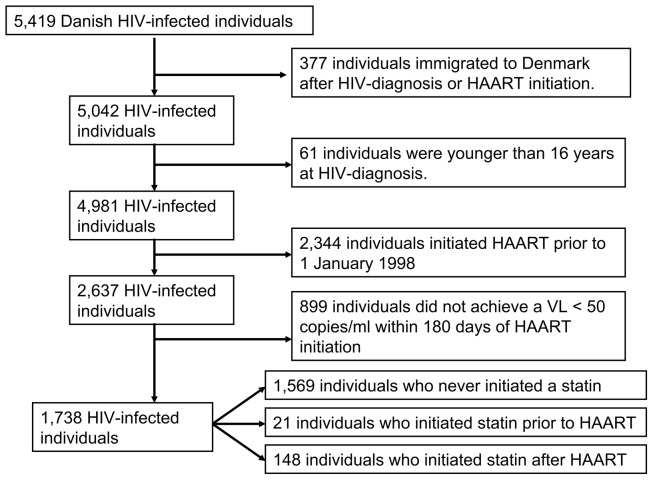
Summary of the study design.

From DHCS we included all Danish HIV positive patients older than 16 years at HIV diagnosis, who started HAART on 1 January 1998 or thereafter and before 31 December 2009, and within 6 months of that date had an undetectable VL (<50 copies/ml). Individuals who immigrated to Denmark after HAART initiation were excluded (Figure 1). The index date was defined as the date of HAART initiation. HAART was defined as a treatment regimen of at least three antiretroviral drugs or a treatment regimen including a combination of a non-nucleoside reverse transcriptase inhibitor and a boosted protease inhibitor and/or integrase inhibitor. Structured treatment interruptions have generally not been used in Denmark, why an individual who initiated HAART was considered on HAART for the rest of the observation period.

### Outcome

The primary outcome was time to death from any cause as registered in the Danish Civil Registration System (DCRS).

### Exposure

Date of first redemption of a prescription of a statin was included as a time-updated covariate. The following statins were included in the analysis: simvastatin, lovastatin, pravastatin, fluvastatin, atorvastatin, cerivastatin, rosuvastatin and a combination of the drugs simvastatin and ezetimibe (Anatomical Therapeutic Chemical Classification code (ATC): C10AA01-07, C10BA02). Statins are reimbursable and only available on prescription in Denmark. They are used according to national guidelines [32] that are almost identical to the ESC/EAS Guidelines [33]. Names of and ATC codes for statins are further provided in the appendix S1. As with HAART, an individual who initiated a statin was considered on statins for the rest of the observation period.

### Covariates and confounder control

We first introduced the following covariates to control for potential confounding (adjustment 1): age (included as a time-updated variable in the following age-intervals 0–29, 30–39, 40–49, 50–59, 60–69, 70+ years), gender, race (Caucasian/non-Caucasian), HIV-transmission group (men who have sex with men (MSM), heterosexual contacts, intravenous drug abuse (IDU), other), hepatitis C status (HCV – defined as being seropositive for HCV and/or having a positive HCV RNA registered in DHCS), calendar year of HAART initiation (<2004 vs. > = 2004), one or more AIDS defining illnesses prior to HAART (yes/no), ART use before initiating HAART, CD4 cell count within +/−1 year of HAART initiation (<50, 50–200, >200cells/µl), HIV viral load (VL) +/−1 year of HAART initiation (log10(VL copies/ml)) and a cholesterol level before or up to 1 year after HAART initiation (<5, 5–8, >8 mmol/L). We further used an extended model (adjustment 2) in which comorbidity and the interaction term between statin use/non-use and comorbidity was also included. In these analyses comorbidity was defined as the date an individual was first diagnosed with one of the following comorbid conditions (coronary artery disease, cerebrovascular disease, peripheral artery disease, chronic kidney disease or a redeemed prescription of an antidiabetic drug (surrogate marker of diabetes mellitus)) as defined in DNHR and DNPR, and introduced as time-updated variable. ICD8, ICD10 and ATC codes are provided in appendix S2 and S3.

### Statistical analysis

We computed time from HAART initiation until date of death from any cause, emigration, lost to follow up or 31 December 2009, whichever occurred first. Table 1 presents a summary of the models used in the study. We first performed the analyses in accordance with the study by Moore et al (model A) in which individuals who developed virological failure were censored at first date of a VL measurement higher than 500 copies/ml after date of first achieved undetectable VL (<50copies/ml). Subsequently a second analysis (model B) was performed with no censoring due to virological failure. We computed mortality rates (MR) per 1,000 person years and used Poisson regression analysis to compute aMRR, as a measure of the relative risk, and 95% confidence intervals (CI). To evaluate the impact of statins on rate of death we included date of statin initiation as a time-updated variable as described above and compared the rate of death before statin initiation to the rate after statin initiation. All analyses were adjusted for potential confounding factors as mentioned above. Due to a clinical important interaction between statin use/non-use and comorbidity, MRRs were estimated for time before or with no comorbidity and after a diagnosis of comorbidity.

**Table 1:** Models used in the study for HIV-infected individuals who initiated HAART 1 January 1998 or thereafter, and within 6 months of that date had an undetectable VL (<50 copies/ml).

Model	Censoring	Adjustment
**A1 (Model A, Adjustment 1)**	Censored at first VL >500 copies****/ml after first VL <50 copies/ml	Age intervals (time-updated), gender, race, HIV-transmission group, hepatitis C status, calendar year of HAART initiation, AIDS defining illnesses prior to HAART, ART use before initiating HAART, CD4 cell count, viral load and cholesterol at HAART initiation.
**A2 (Model A, Adjustment 2)**	As for A1	As model A1, but also including first date of comorbidity (cardiovascular disease, chronic kidney disease and diabetes) as time updated covariate and the interaction term between statin use/non-use and comorbidity
**B1 (Model B, Adjustment 1)**	Not censored at****virological failure	As for A1
**B2 (Model B, Adjustment 2)**	As for B1	As for A2

SPSS version 19.0 (SPSS Inc., Chicago, Illinois, USA) and STATA software, version 11.0 (Stata Corporation, college Station Texas, USA) were used for data analyses. Data from DNHR and DNPR was obtained with approval from the Danish Registry Board. The study was approved by the Danish Data Protection Agency (record number 2008-41-1781).

## Results

Figure 1 presents a summary of the study design. The study cohort consisted of 1,738 HIV-infected individuals who initiated HAART on 1 January 1998 or after this date and within 180 days of that had a VL <50 copies/ml. Two main analyses were performed (table 1). In the first analysis (model A) time of follow-up was censored in 396 (22.8%) individuals at date of virological failure (VL >500 copies/ml). In the second analysis (model B) individuals were not censored due to virological failure.

In model A, 145 (8.3%) HIV-infected individuals initiated a statin of whom 124 (7.1%) were started after HAART initiation. These analyses gave rise to a total of 7,952 person-years of follow-up (PYR). Of these 7,528 PYR were before and 424 PYR after statin initiation. In total 109 (6.3%) individuals died of which 7 (6.4%) had initiated a statin drug prior to death. In Model B 169 (9.7%) individuals initiated a statin (148 (8.5%) after HAART initiation) and had a total of 9,865 PYR (9,358 PYR before and 506 PYR after statin initiation). 171 HIV-infected individuals died, of whom 15 had initiated a statin. Additional characteristics of the HIV-infected individuals are provided in table 2. The generic types of statin initiated were simvastatin (53.3%), pravastatin (31.2%), atorvastatin (5.9%) and rosuvastatin (9.5%).

**Table 2:** Characteristics of HIV**-**infected individuals initiating HAART after 1 January 1998 with a VL < 50 copies/ml within 180 days of HAART initiation.

	All HIV-infected individuals initiating HAART after 1 January 1998 with a VL < 50 copies/ml within 180 days of HAART initiation		
	All	Individuals with Non-STATIN time	Individuals with time on a STATIN
	(N = 1,738)	(N = 1,717)	(N = 169)
**Male gender, N (%)**	1,270 (73.1)	1,252 (72.9)	141 (83.4)
**Caucasian race, N (%)**	1,340 (77.1)	1,322 (77.0)	151 (89.3)
**Infection mode:**			
** MSM, N (%)**	761 (43.8)	754 (43.9)	88 (52.1)
** Heterosexual contact, N (%)**	728 (41.9)	715 (41.6)	70 (41.4)
** Intravenous drug abuse, N (%)**	154 (8.9)	154 (9.0)	3 (1.8)
** Other/unknown, N (%)**	95 (5.5)	94 (5.5)	8 (4.7))
**Hepatitis C infection, N (%)**	254 (14.6)	254 (14.8)	6 (3.6)
**No of patients with 1 or more AIDS defining event prior to HAART initiation, N (%)**	301 (17.3)	300 (17.5)	33 (19.5)
**HAART initiated prior to year 2004, N (%)**	914 (52.6)	911 (53.1)	119 (70.4)
**Initiated ART before HAART, N (%)**	140 (8.1)	138 (8.0)	17 (10.1)
**CD4 cell count at HAART initiation, cells/µl**			
** 0-50**	228 (13.1)	228 (13.3)	26 (15.4)
** > 50-200**	502 (28.9)	498 (29.0)	45 (26.6)
** >200**	1,008 (58.0)	991 (57.7)	98 (58.0)
**HIV VL at HAART initiation, median log10(VL copies/ml)(IQR)**	4.8 (4.1-5.3)	4.8 (4.1-5.3)	4.8 (4.3-5.2)
**Nadir CD4 cell count, median cells/µl (IQR)**	190 (90-260)	196 (97-266)	191 (86-255)
**Total cholesterol at HAART initiation, mmol/L**			
** < 5**	1,003 (57.7)	989 (57.6)	68 (40.2)
** 5-8**	481 (27.7)	477 (27.8)	77 (45.6)
** >8**	11 (0.6)	10 (0.6)	6 (3.6)
** Missing data**	243 (14.0)	241 (14.0)	18 (10.7)

	CENSORED AT DATE OF VIROLOGICAL FAILURE		
	(VL > 500 copies/ml) (Model A)		
	All	Individuals with Non-STATIN time	Individuals with time on a STATIN
	(N = 1,738)	(N = 1,717)	(N = 145)
**Age at start of statin/non-statin period, median years (IQR)**	39.3 (33.0-46.5)	39.2 (33.0-46.3)	53.3 (46.9-60.3)
**Comorbidity:**			
** One or more comorbid conditions before** ** index date, N (%)**	82 (4.7)	67 (3.9)	59 (40.7)
** One or more comorbid conditions after** ** index date, N (%)**	89 (5.1)	76 (4.4)	13 (9.0)
**Duration of follow-up, person-years**	7,952	7,528	424
**Duration of follow-up, median-years (IQR)**	3.7 (1.5-7.3)	3.5 (1.5-6.9)	2.4 (1.0-4.1)
** Emigration during follow-up, N (%)**	24 (1.4)	23 (1.3)	1 (0.7)
** Lost to follow-up, N (%)**	2 (0.1)	2 (0.1)	0 (0)
**Initiated a statin drug before HAART initiation, N (%)**	21 (1.2)	0 (0)	21 (14.5)
**Initiated a statin drug after HAART initiation, N (%)**	124 (7.1)	[124 (7.2)] = >	124 (85.5)
**Duration of follow-up without statin, person-years**	7,528	7,528	-
**Duration of follow-up with statin, person-years**	424	-	424
**Total no. of deaths, N (%)**	109 (6.3)	102 (5.9)	7 (4.8)
** Age at death, median years (IQR)**	53.6 (44.5-62.4)	53.3 (61.8-44.0)	64.2 (59.0-68.4)
** Initiated a statin drug prior to death, N (%)**	7 (6.4)	0 (0)	7 (100)
**MR per 1,000 person years (95%CI)**	13.7(11.4-16.5)	13.5(11.2-16.5)	16.5(7.9-34.7)

	NOT CENSORED AT VIROLOGICAL FAILURE		
	(VL > 500 copies/ml) (Model B)		
	(N = 1,738)	(N = 1,717)	(N = 169)
**Age at start of statin/non-statin period, median years (IQR)**	39.3 (33.0-46.5)	39.2 (33.0-46.3)	52.9 (45.5-60.4)
**Comorbidity:**			
**One or more comorbid conditions before index date, N (%)**	82 (4.7)	67 (3.9)	71 (42.0)
**One or more comorbid conditions after index date, N (%)**	121 (7.0)	106 (6.2)	15 (8.9)
**Duration of follow-up, person-years**	9,865	9,358	506
**Duration of follow-up, median-years (IQR)**	5.6 (2.5-8.7)	5.3 (2.4-8.3)	2.5 (1.2-4.1)
** Emigration during follow-up, N (%)**	32 (1.8)	31 (1.8)	1 (0.6)
** Lost to follow-up, N (%)**	6 (0.3)	6 (0.3)	0 (0)
**Initiated a statin drug before HAART initiation, N (%)**	21 (1.2)	0 (0)	21 (12.4)
**Initiated a statin drug after HAART initiation, N (%)**	148 (8.5)	[148 (8.6)] = >	148 (87.6)
**Duration of follow-up without statin, person-years**	9,358	9,358	-
**Duration of follow-up with statin, person-years**	506	-	506
**Total no. of deaths, N (%)**	171 (9.8)	156 (9.1)	15 (8.9)
**Age at death, median years (IQR)**	50.5 (43.4-61.6)	49.3 (42.7-60.4)	63.1 (59.0-67.6)
**Initiated a statin drug prior to death, N (%)**	15 (8.8)	0	15 (100)
**MR per 1,000 person years (95%CI)**	17.3(14.9-20.1)	16.7(14.2-19.5)	29.6(17.9-49.1)

**Abbreviations:** IQR: Interquartile range; MR: Mortality Rate; 95% CI: 95% Confidence Interval; MSM: men who have sex with men; HAART: Highly Active Antiretroviral Therapy; VL: Viral Load.

* The column “Individuals with Non-STATIN time” includes data on all patients who were not on statin treatment at study entry, and the column “Individuals with time on a STATIN” includes data on all patients who during observation time were treated with statin. Some patients were therefore included in both columns why the total number adds up to more than 100%.

For conversion from the SI unit mmol/L to mg/dL: (mg/dL cholesterol  =  mmol/L * 38.6) [33].

In the analysis confined to observation time without virological failure (model A1) we found an aMRR of 0.75 (95% CI: 0.33–1.68) for the time after versus the time before or with no initiation of a statin (Table 3). In the analysis with no censoring at virological failure (model B1) the aMRR was 1.17 (95% CI 0.66–2.07) (Table 4). In line with prior knowledge, use of statin seemed to reduce the rate of death in HIV-infected individuals after a diagnosis of comorbidity in both models {( model A 2: aMRR 0.34; 95% CI: 0.11–1.04), ( model B2: 0.64; 95% CI: 0.32–1.29), but no difference could be detected in the time before or without such a diagnosis {( model A2: aMRR 1.12; 95% CI: 0.34–3.62, model B2: aMRR: 0.90; 95% CI: 0.28–2.88)} (Table 3 and 4).

**Table 3:** Mortality rate ratio (MRR) of HIV-infected individuals initiating HAART after 1 January 1998 with a VL <50 copies/ml within 180 days of HAART initiation with censoring of individuals with virological failure (VL >500copies/ml) comparing time on statin with time not on statin.

	CENSORED AT DATE OF VIROLOGICAL FAILURE				
		(VL >500 copies/ml) (Model A)			
				MRR (95%CI)	
STATIN USE (time-updated variable)	No. of Deaths	PYR	MR per 1,000 PYR (95% CI)	Unadjusted	Adjusted
**Adjustment 1:** *					
**No use of or time before initiation of a statin drug**	102	7,528	13.55 (11.16–16.45)	Ref (1)	Ref (1)
**Use of or time after initiation of a statin drug**	7	424	16.52 (7.88–34.66)	1.22 (0.57–2.62)	0.75 (0.33–1.68)
**Adjustment 2:** *					
**BEFORE OR NO DIAGNOSIS OF COMORBIDITY:**					
**No use of or time before initiation of a statin drug**	84	7,138	11.77 (9.50–14.57)	Ref (1)	Ref (1)
**Use of or time after initiation of a statin drug**	3	184	16.33 (5.27–50.62)	1.39 (0.44–4.39)	1.12 (0.34–3.62)
**AFTER A DIAGNOSIS OF COMORBIDITY:**					
**No use of or time before initiation of a statin drug**	18	390	46.11 (29.05–73.19)	Ref (1)	Ref (1)
**Use of or time after initiation of a statin drug**	4	240	16.67 (6.26–44.42)	0.36 (0.12–1.07)	0.34 (0.11–1.04)

**Abbreviations:** MRM: Mortality Rate Ratio; 95% CI: 95% Confidence Interval; PYR: Person years of follow-up; MSM: men who have sex with men; HAART: Highly Active Antiretroviral Therapy.

* **Adjustment 1:** Adjusted for age (treated as time-updated variables split at 30, 40, 50, 60,70), gender, race, HIV transmission group, CD4 cell count at HAART initiation (<50, 50–200, >200 cells/µl), HIV VL at HAART initiation (log_10_ VL), total cholesterol (<5, 5–8, >8, missing values), year of HAART initiation (<2004 vs. > = 2004), ART prior to HAART, AIDS defining illness prior to HAART initiation, Viral hepatitis C co-infection.

* **Adjustment 2:** Adjusted for variables as in model 1 + comorbidity and the clinically important interaction between comorbidity and statin use/non-use, The MRRs are therefore presented both before and after development of a comorbid condition. Comorbidity is defined as the first of the following comorbid conditions: coronary artery disease, cerebrovascular disease, peripheral artery disease, chronic kidney disease and a redeemed prescription of an antidiabetic drug and introduced as a time-updated variable.

**Table 4:** Mortality rate ratio (MRR) of HIV-infected individuals initiating HAART after 1 January 1998 with a VL <50copies/ml within 180 days of HAART initiation, with no censoring due to virological failure, comparing time on statin with time not on statin.

	NOT CENSORED AT DATE OF VIROLOGICAL FAILURE				
	(VL >500 copies/ml) (Model B)				
				MRR (95%CI)	
STATIN USE (time-updated variable)	No. of Deaths	PYR	MR per 1,000 PYR (95% CI)	Unadjusted	Adjusted
* **Adjustment 1:**					
**No use of or time before initiation of a statin drug**	156	9.358	16.67 (14.25–19.50)	Ref (1)	Ref (1)
**Use of or time after initiation of a statin drug**	15	506	29.62 (17.86–49.13)	1.78 (1.05–3.02)	1.17 (0.66–2.07)
* **Adjustment 2:**					
**BEFORE OR NO DIAGNOSIS OF COMORBIDITY:**					
**No use of or time before initiation of a statin drug**	126	8,853	14.23 (11.95–16.95)	Ref (1)	Ref (1)
**Use of or time after initiation of a statin drug**	3	223	13.49 (4.35–41.81)	0.95 (0.30–2.98)	0.90 (0.28–2.88)
**AFTER A DIAGNOSIS OF COMORBIDITY:**					
**No use of or time before initiation of a statin drug**	30	506	59.33 (41.48–84.85)	Ref (1)	Ref (1)
**Use of or time after initiation of a statin drug**	12	284	42.26 (24.00–74.42)	0.71 (0.36–1.39)	0.64 (0.32–1.29)

**Abbreviations**: MRM: Mortality Rate Ratio; 95% CI: 95% Confidence Interval; PYR: Person years of follow-up; MSM: men who have sex with men; HAART: Highly Active Antiretroviral Therapy.

* **Adjustment 1**: Adjusted for age (treated as time-updated variables split at 30, 40, 50, 60,70), gender, race, HIV transmission group, CD4 cell count at HAART initiation (<50, 50–200, >200 cells/µl), HIV VL at HAART initiation (log10 VL), total cholesterol (<5, 5–8, >8, missing values), year of HAART initiation (<2004 vs. > = 2004), ART prior to HAART, AIDS defining illness prior to HAART initiation, Viral hepatitis C co-infection.

* **Adjustment 2:** Adjusted for variables as in model 1+ comorbidity and the clinically important interaction between comorbidity and statin use/non-use, The MRRs are therefore presented both before and after development of a comorbid condition. Comorbidity is defined as the first of the following comorbid conditions: coronary artery disease, cerebrovascular disease, peripheral artery disease, chronic kidney disease and a redeemed prescription of an antidiabetic drug and introduced as a time-updated variable.

## Discussion

Despite higher rates of comorbidity (cardiovascular disease, chronic kidney disease and diabetes) in HIV-infected statin users, we observed a trend towards a reduction in all-cause mortality in association with statin therapy in the analyses where patients with virological failure (model A) were censored. In analyses with no censoring (model B), statin users died at the same rate as non-users. Although, use of statin reduced mortality in individuals after a diagnosis of comorbidity the impact on individuals with no comorbidity seemed minimal or absent.

The strengths of our study include use of a nationwide population-based cohort with a long observation period and complete follow-up. As we had access to Danish registries of a high quality we could identify all redeemed prescriptions on statins dispensed at Danish community pharmacies as well as valid data on date of death. Furthermore, the availability of electronically collected data on VL, CD4 cell counts and history of antiretroviral treatment from DHCS, minimized potential selection and information bias. As the main aim of the study was to investigate whether we could confirm the findings by Moore et al. [23], we conducted the analysis using model A1 (Table 1) with only minor differences compared to their study. However, model A might have been biased by informed censoring [34], why model B was conducted. Both model A1 and B1 might have been biased by confounding by indication [24]. We therefore made additional analyses adjusted for comorbidity treated as a time-updated variable and included the clinically important interaction term between comorbidity and statin-use/non-use in the model (model A2 and B2).

Our study has some limitations. We did not consider the specific type of statin therapy or the degree of exposure (dosage and duration), thus assuming that statin-associated benefits were a class effect. However, as statins possess different potencies for HMG CoA reductase inhibition, due to differences in tissue permeability and metabolism this could lead to differences between studies. We analyzed the redemption of prescriptions of statins given the assumption that drug acquisition was a reasonable surrogate for consumption. However, adherence problems might exist. Furthermore, we did not consider cessations or changes of either statin or HAART. In the multivariate analyses we included the baseline CD4 cell count, viral load and cholesterol level; however, we are aware that the absence of the dynamic of these parameters on HAART could have affected the results. Furthermore, as we had no dynamic data on high-density lipoprotein, smoking status, body mass index or blood pressure, we could not adjust for these potential confounders. Due to the study design, we had to rely on hospital registry-based discharge diagnoses in order to identify comorbidity. Importantly, we used the same source of data to ascertain comorbidity for all study subjects. As registration of diagnoses is restricted to hospital contacts, we used first date of redemption of an antidiabetic drug as a surrogate marker of diabetes, as ICD codes are not valid for identification of diabetes. If individuals under study are unlikely to obtain the prescribed medication from sources not captured by the database, the measure can be considered to have a high specificity [31]. A recent study [35] validated the diagnostic codes (ICD-10) used to ascertain the Charlson comorbidity index against the diagnoses assigned by the treating physician and found a consistently high positive predictive value (PPV) that was above 95% for myocardial infarction, peripheral artery disease, cerebrovascular disease and chronic kidney disease. Angina pectoris, for which the validity may be somewhat lower, was however not included in this analysis. Furthermore, information on patients with comorbidity that was not diagnosed at the hospital, as well as prehospital death was not covered. Other studies have found high [36] to moderate PPV [37]–[38], however, these studies were meant to validate the diagnoses assessed by the treating physician. Finally, despite adjustments for a number of confounders, we cannot exclude bias due to unmeasured and residual confounding.

In a recent study Moore et al. [23] found that in HIV-infected individuals statin therapy was associated with a statistically significant 3 fold reduction in mortality (adjusted hazard ratio (aHR):0.33; 95% CI: 0.14–0.76). We were unable to reproduce this highly significant reduction in mortality. We presume some methodological problems might bias the results by Moore et al. [23]. Although we conducted model A almost as done in the study by Moore et al. [23], there are some differences that potentially could contribute to the difference in results. First, accessibility to the healthcare service (hospital and general practitioner) in Denmark is quite high and free of charge. Moreover, as statins are reimbursable, the prescriptions of these drugs rely mainly on an objective risk assessment [32]–[33] and are not affected by the quality of health care insurances. Second, the Johns Hopkins HIV clinical cohort, used by Moore et al [23], consists of patients who presents themselves for HIV care at the institutions and agree to participate in the cohort study, whereas our cohort is population based and nationwide. Third, baseline characteristics of our HIV-infected population differed from that used by Moore et al. [23] (fever females, more Caucasians, less IDUs, less HCV). Also the nadir CD4 cell count, which is a marker for poor clinical outcome, might differ between groups. Fourth, the median time of follow-up was substantially shorter in the study by Moore et al. [23] (1.6 years (median time on statin: 2 years)) than in our study (Model A) and fewer people died (85 in total, 7 on statin, 78 not on statin). This could rely on differences in the amount of individuals censored; however, the latter data is not accessible in the study by Moore et al. [23]. Fifth, the type of statin used differed largely as the indication for prescribing statin (primary/secondary prophylaxis) might have done. Sixth, Moore et al. [23] required that patients had to receive statin for at least 30 days; however, as both HAART and statin use were based on prescribing those drugs and not the filling of a prescription, adherence problems could be a larger issue than in our study. Seventh, Moore et al. [23] censored follow-up in individuals with virological failure. This could seem like a sensible method to control for confounding, but as indicated above, it could lead to serious bias. We applied an additional strategy (model B), in which time was not censored at virological failure, and found no difference in mortality in association with statin therapy. Although model B would not be affected by informative censoring, there may however be a substantial amount of patient follow-up time where a non-suppressed viral load, which is strongly associated with increased mortality, could result in bias towards the null. Eighth, Moore et al. [23] adjusted the analysis for a number of important covariates including hemoglobin. Although hemoglobin has been found to be a strong independent prognostic marker of death [39], we presume that the lack of this covariate in our analysis cannot explain the difference in results. Ninth, Moore et al. [23] did not take effects of comorbidity into account.

In an unpublished ACTG study, presented at the 19^th^ Conference on Retroviruses and opportunistic infections (CROI), 2011, Overton et al. reported reduced risk of the composite endpoint serious non-AIDS events/death (aHR: 0.81; 95%CI: 0.53–1.24) and malignancies (aHR 0.43; 95% CI: 0.19–0.94) in patients who were treated with statin.

The reduced mortality after statin initiation found by Moore et al. [23] and in our study (model A1) might rely on confounding from several factors such as alcohol consumption, smoking, obesity and non-AIDS morbidity (cardiovascular as well as non-cardiovascular). Also factors that might affect the allocation of a patient to statin therapy such as ethnicity, abuse, compliance and health seeking behavior in general may confound the results. In some studies the association between statin use and adverse outcomes, has been proposed to be due to a healthy user/adherer effect [40]–[41]. Initiation of and adherence to statin therapy could therefore be a surrogate marker for a higher medical attention and a healthier lifestyle [40]–[42]. We cannot exclude that these factors could bias our study, the study by Moore et al. [23] and Overton et al. [39], in which case the effect of statin would be overestimated. Despite major focus on cardiovascular disease in HIV-infected individuals, individuals not on statin might have unrecognized heart disease or risk factors. Furthermore, as the indications for the use of statins have been expanded during the last decade high-risk patients might have been substantially under-treated in the early years [1], [8].

In a large meta-analysis of 14 RCTs of statin effects (90,056 individuals with cardiovascular disease, diabetes or risk factors) [9], Baigent et al. found a significant reduction in all-cause mortality and death due to vascular diseases but no difference regarding non-vascular causes of death between statin users and non-users (RR 0.95, 95%CI 0.91–1.01). In line with this we found very little if any impact of statin therapy on overall mortality in the time before or with no diagnosis of comorbidity (model A2 and B2). As our analysis did not have the power to address subgroups of individuals with e.g. immunologic non-response (i.e. persistently low CD4 cell counts despite years of suppressive HAART), we cannot exclude that statins might be effective for a subgroup of patients with substantially elevated levels of immune activation.

Given the observational study design, an RCT of an appropriate size is needed to achieve a valid evaluation of statin effects on mortality in HIV-infected individuals. However, using the mortality rates seen in our study, more than 25.000 patients have to be recruited into each arm in an RCT to detect a 10% reduction in mortality with 3 years of follow-up (alpha: 0.05, power: 0.80). Statins are already widely used and although these drugs are rather safe and tolerable, 3 recent meta-analyses [43]–[45] have found that statin therapy might be associated with a modest, dose-dependent, 9–12% higher risk of new-onset diabetes mellitus. The clinical importance of this potential risk seems to be outweighed by the cardiovascular benefit in individuals for whom statin therapy is recommended, [43]–[45]. But, if new indications are emerging, in which statin therapy are used for patients at low cardiovascular risk, the risk might not outweigh the benefits [45].

In conclusion, statin therapy might have a beneficial effect on all-cause mortality in HIV-infected individuals, but the impact in individuals with no cardiovascular disease, chronic kidney disease or diabetes is small or absent. An RCT is needed to make an evidence-based proof of a causal relation. However, as patients with high risks of cardiovascular disease obviously cannot be included in the trial, the sample size needed may be prohibitive for the conduct of the study.

## Supporting Information

Appendix S1 **ATC codes of cholesterol reducing drugs.**
(DOC)

Appendix S2 **Diagnostic codes of comorbidity (ICD 8 and ICD 10 codes).**
(DOC)

Appendix S3 **ATC codes of antidiabetic drugs.**
(DOC)
