# Supplementary material for: Statin Therapy and Mortality in HIV-Infected Individuals; A Danish Nationwide Population-Based Cohort Study
Source: PLoS One. 2013 Mar 4;8(3):e52828. doi: 10.1371/journal.pone.0052828 (PMC3587599; doi:10.1371/journal.pone.0052828)
Supplement: Appendix S1 — ATC codes of cholesterol reducing drugs. (DOC) [file pone.0052828.s001.doc]

APPENDIX S1:

**CHOLESTEROL REDUCING DRUGS (STATINS):**

(ATC=Anatomical Therapeutic Chemical Classification)

**STATINS:**

C10AA01: Simvastatin

C10AA02: Lovastatin

C10AA03: Pravastatin

C10AA04: Fluvastatin

C10AA05: Atorvastatin

C10AA06: Cerivastatin

C10AA07: Rosuvastatin

**COMBINATION DRUGS:**

C10BA02: Simvastatin and ezetimibe

The ATC code as defined in the 2010 index is established through a linkage to the World Health Organization’s Collaboration Centre for Drug Statistics Methodology [“ATC-index” WHO Collaborating Centre for Drug Statistics Methodology. Norwegian Institute of Public Health. Available at: [<http://www.whocc.no/atc_ddd_index/>]].

The ATC-codes: B04AB01-04 (Simvastatin, lovastatin, pravastain and fluvastatin) were changed: January 1, 1997: to C10AA01-C10AA04 .
