# Supplementary material for: Statin Therapy and Mortality in HIV-Infected Individuals; A Danish Nationwide Population-Based Cohort Study
Source: PLoS One. 2013 Mar 4;8(3):e52828. doi: 10.1371/journal.pone.0052828 (PMC3587599; doi:10.1371/journal.pone.0052828)
Supplement: Appendix S2 — Diagnostic codes of comorbidity (ICD 8 and ICD 10 codes). (DOC) [file pone.0052828.s002.doc]

APPENDIX S2:

**DIAGNOSTIC CODES OF COMORBIDITY:**

(Both primary and secondary diagnoses were used in the analyses)

**CORONARY ARTERY DISEASE:**

**ICD 8:** 410.09-414.99

**ICD 10:** I20.0-25.9

**CEREBROVASCULAR DISEASE:**

**ICD8:** 430.00-438.99

**ICD10:** I60.0-69.8, G45.0-46.8

**PERIPHERAL ARTERY DISEASE:**

**ICD 8:** 440.09-440.99

**ICD 10:** I70.0-70.9

**CHRONIC KIDNEY DISEASE:**

**ICD 8:** 580.00, 582.00-584.99, 593.20

**ICD 10:** N02.0-02.8, N03.0-03.9, N05.0-6.8, N07.0-8.8, N14.0-14.4, N18.0-18.9, I12.0
