# Supplementary material for: Statin Therapy and Mortality in HIV-Infected Individuals; A Danish Nationwide Population-Based Cohort Study
Source: PLoS One. 2013 Mar 4;8(3):e52828. doi: 10.1371/journal.pone.0052828 (PMC3587599; doi:10.1371/journal.pone.0052828)
Supplement: Appendix S3 — ATC codes of antidiabetic drugs. (DOC) [file pone.0052828.s003.doc]

APPENDIX S3:

**ANTIDIABETIC DRUGS:**

(ATC=Anatomical Therapeutic Chemical Classification)

**INSULIN AND ANALOGUES FOR INJECTION, FAST-ACTING:**

A10AB01: Insulin (human)

A10AB04: Insulin lispro

A10AB05: Insulin aspart

A10AB06: Insulin glulisine

**A10AC: INSULINS AND ANALOGUES FOR INJECTING, INTERMEDIATE ACTING**

A10AC01: Insulin (human)

**INSULINS AND ANALOGUES FOR INJECTING, INTERMEDIATE ACTING COMBINED WITH FAST-ACTING**

A10AD01: Insulin (human)

A10AD04: Insulin lispro

 A10AD05: Insulin aspart

**INSULINS FOR INJECTION, LONG ACTING:**

A10AE01: Insulin (human)

A10AE04: Insulin glargine

A10AE05: Insulin detemir

The ATC code as defined in the 2010 index is established through a linkage to the World Health Organization’s Collaboration Centre for Drug Statistics Methodology [“ATC-index” WHO Collaborating Centre for Drug Statistics Methodology. Norwegian Institute of Public Health. Available at: [<http://www.whocc.no/atc_ddd_index/>]].

The ATC-codes A10AA01-04 (Insulin and analogues – fast-acting, long acting, combination therapy and long acting) were changed 1 January 1997 to the ATC-codes A10AB, A10AC, A10AD, A10AE.

**ORAL ANTIDIABETIC DRUGS**

**A10BA:BIGUANUIDES**

A10BA02: Metformin

**A10BB: SULFONAMIDES, UREA DERIVATES**

A10BB01: Glibenclamide

A10BB03: Tolbutamide

A10BB04: Glibornuride

A10BB07: Glipizide

A10BB09: Gliclazide

A10BB12: Glimepiride

**COMBINATIONS OF ORAL BLOOD GLUCOSE LOWERING DRUGS**

A10BD03: Metformin and rosiglitazone

A10BD04: Glimepiride and rosiglitazone

A10BD07: Metformin and sitagliptin

A10BD08: Metformin and vildagliptin

**ALPHA GLUCOSIDASE INHIBITORS:**

A10BF01: Acarbose

**THIAZOLODINEDIONES:**

A10BG02: Rosiglitazone

A10BG03: Pioglitazone

**DIPEPTIDYL PEPTIDASE 4 (DPP-4) INHIBITORS:**

A10BH01: Sitagliptin

A10BH02: Vildagliptin

A10BH03: Saxagliptin

**OTHER BLOOD GLUCOSE LOWERING DRUGS, EXCLUDING INSULINS:**

A10BX02: Repaglinide

A10BX04: Exenatide

A10BX07: Liraglutide
